# Status of Contraceptive Use for Birth Spacing After a Teenage Pregnancy: Where Do We Stand?

**DOI:** 10.7759/cureus.38563

**Published:** 2023-05-04

**Authors:** Anupma A, Avir Sarkar, Priyanka Sharma, Sonam Jindal, Jagadish C Sharma

**Affiliations:** 1 Obstetrics and Gynecology, Employee State Insurance Corporation (ESIC) Medical College and Hospital, Faridabad, Faridabad, IND

**Keywords:** family planning, postpartum contraception, contraception, teenage pregnancy, adolescent pregnancy

## Abstract

Background: Adolescence is the most complex stage of reproductive health. The knowledge and awareness of adolescent-related reproductive issues are limited, particularly in lower-middle-income countries. Adolescent pregnancies are associated with major maternal and neonatal complications. Effective contraception use can prevent teenage pregnancy and subsequent complications.

Methods: It was a cross-sectional study conducted in a tertiary care hospital and teaching institute over a period of one year. Through this study, we aimed to assess the prevalence of postpartum use of approved standard methods of contraception for birth spacing among teenage mothers and to assess the reasons for their non-acceptance. A total of 133 consecutive consenting postpartum teenage mothers were recruited in the study. Participants were asked about their age at the time of marriage and delivery, marital status, parity, education and economic status, the number of antenatal visits, mode of delivery, and antenatal complications. Compliance with postpartum contraception was noted, and reasons for its non-acceptance were asked in detail.

Results: Among the 133 participants, contraceptive users were categorized into Group A and non-users into Group B. The mean age at the time of marriage was 17.6±0.4 and 17.5±0.6 years in Group A and Group B, respectively. Mothers in Group A were more educated than their counterparts in Group B (82.2% of mothers were educated up to 12th standard in Group A compared to 46.6% in Group B). Among the contraception users, 70% had four or more antenatal visits compared to 7.9% of the non-users. Reasons for non-acceptance of postpartum contraception were elicited in Group B: 42.0% had fear of becoming infertile, 38.6% feared that contraceptives interfere with breastfeeding and quality of breastmilk, 13.6% had opposition from family members, and 5.8% did not mention any reason.

Conclusion: Teenage pregnancy is associated with increased feto-maternal complications. It also accounts for an increased incidence of unsafe abortions and maternal mortality. So it is crucial to make the adolescent group aware of effective methods of postpartum contraceptives to prevent adolescent pregnancies. Collaborative larger multicentric studies from different countries will help to reach a better, generalized conclusion regarding the same.

## Introduction

Adolescent pregnancies are associated with major maternal and neonatal complications. A prospective study on antenatal teenage mothers showed that the majority of the pregnancies were complicated by anemia [1]. Adolescence is one of the most complex stages of reproductive health. The knowledge and awareness of these adolescent-related reproductive issues are limited, particularly in lower-middle-income countries (LMIC) [2]. Effective use of approved methods of contraception can prevent teenage pregnancy and its sequelae. A study involving middle and high school students in the United States showed that 31.6% of female participants were less likely to use contraception compared to their male counterparts [3]. There exists a poor awareness and acceptance of postpartum contraception among the vulnerable adolescent population, which leads to unintended pregnancies with short inter-conception periods among married teenage couples in the LMIC. Thus it is very important to assess the factors leading to poor acceptance of postpartum contraception among adolescent mothers so that Quality Improvement (QI) initiatives can be taken in the future.

## Materials and methods

It was a cross-sectional study conducted in a tertiary care hospital and teaching institute (Employee State Insurance Corporation {ESIC} Medical College and Hospital, Faridabad) over a period of one year. Informed consent was obtained from all participants. Institutional Ethics Committee approval was obtained from ESIC Medical College and Hospital, Faridabad (134/X/11/13/2021-IEC/36) prior to the commencement of the study. All procedures in the study involving human participants were performed in accordance with the ethical standards of the 1964 Helsinki Declaration and its later amendments or comparable ethical standards. All teenage mothers who attended postpartum clinics to seek maternal and child health care services during the study time frame were potentially eligible for recruitment. The research question aimed to assess the prevalence of acceptance of standard methods of postpartum contraception among adolescent mothers and to study the factors leading to their non-acceptance, if any. Inclusion criteria consisted of women less than 20 years of age coming for routine postnatal visits and consenting to participate in the study. Adolescent mothers aged between 14 and 19 years, who had given birth within one year, were eligible for the interview. Young women less than 14 years of age were preferably excluded due to comparability to other international studies. Both married and unmarried girls were proposed to be included in the study.

The legal age of marriage in the study area is 18 years. Although participants aged 18 years and above gave written informed consents, those below 18 years were recruited after obtaining written assent and informed consent from the legal guardian (husband or parents). Mothers not willing to give written informed consent or assent, adolescent pregnancies presenting below 14 years of age, and mothers suffering from chronic mental health disorders and medico-surgical disorders affecting the decision of contraceptive use were excluded from the study. Participants were asked about their marital status, age at the time of marriage and delivery, parity, education, and socio-economic status. Socio-economic status was classified according to the Modified Kuppuswamy Scale, taking education, occupation, and monthly income of the head of the family into consideration. They were asked about the number of antenatal visits, mode of delivery, and antenatal complications in the preceding pregnancy. Compliance with postpartum contraception was noted and reasons for its non-acceptance were asked in detail. All recruited postpartum mothers were subjected to a questionnaire enquiring about the reasons for the non-acceptance of contraception. The questionnaire was self-designed and validated in the first 30 participants with a Cronbach's alpha of 0.74. Once validated, this set of questions was used for all the participants in the study. The questions and consent forms were provided in the vernacular language (Hindi) to ensure that all women understood and responded correctly. 

Taking the previous study by Chuy et al. [3] in consideration and keeping a 95% confidence interval and 80% power of the study, the minimum sample size for the study was calculated to be 102. We could recruit 133 mothers during the study time frame. All data were tabulated in a Microsoft Excel worksheet (Microsoft, Redmond, WA) and statistical analysis was performed by SPSS version 23.0 (IBM Corp., Armonk, NY). All the continuous variables were summarized as means with the corresponding standard deviations (SD), while the categorical variables were summarized as frequencies and percentages. The outcome variable was categorized as use and non-use of any method of contraception based on the participants' report. We used the Chi-square test and Student's t-test to determine the association between the primary outcome and various social demographic and economic factors through bivariate analysis. All factors with a p-value of 0.05 and below were considered statistically significant.

## Results

One hundred and thirty-three postpartum teenage mothers were recruited into the study within the stipulated time frame. All participants were married. Among 133 postpartum teenage mothers, 45 were contraception users (Group A) and 88 were non-users (Group B) (Table 1). The mean age at the time of marriage was 17.6±0.4 and 17.5±0.6 years in Group A and Group B, respectively. The mean age at the time of delivery was 18.4±0.5 and 18.2±0.4 years (Table 1). No difference in parity was observed between contraceptive users and non-users (95.5% were primiparous and 4.5% were multiparous in Group A, 96.6% were primiparous and 3.4% were multiparous in Group B). Women in Group A were more educated compared to their counterparts in Group B (82.2% were educated up to 12th standard in Group A compared to 46.6% in Group B; p-value 0.016), as evident from Table 1. Thus educational status played a pivotal role in the awareness, acceptance, and usage of contraception.

**Table 1:** Table showing the results of the study

S. no.	Characteristics			Contraceptive users (n=45)	Non-users (n=88)	p-value
1.	Mean age at the time of marriage			17.6±0.4	17.5±0.6	0.315
2.	Mean age at the time of delivery			18.4±0.5	18.2±0.4	0.013
3.	Marital status	Married		45(100%)	88(100%)	1.0
		Unmarried		0(0.0%)	0(0.0%)	
4.	Parity	Primigravida		43(95.5%)	85(96.6%)	0.782
		Multigravida		2(4.5%)	3(3.4%)	
5.	Education status	Below fifth standard		8(17.8%)	47(53.4%)	0.016
		Sixth to 12^th^ standard		37(82.2%)	41(46.6%)	
		Graduation		0(0.0%)	0(0.0%)	
6.	Socio economic status	Lower		19(42.3%)	78(88.6%)	0.003
		Middle		26(57.7%)	10(11.4%)	
		Upper		0(0.0%)	0(0.0%)	
7.	Total number of antenatal visits	Less than four		27(60.0%)	81(92.1%)	0.001
		Four and above (at least one in each trimester)		18(40.0%)	7(7.9%)	
8.	Mode of delivery		Vaginal delivery	27(60.0%)	52(59.1%)	0.893
			Caesarean section	18(40.0%)	36(40.9%)	
9.	Antenatal complications		No	6(13.3%)	10(11.4%)	0.445
			Yes	39(86.7%)	78(88.6%)	
10.	Reasons for non-acceptance of postpartum contraception		Fear of becoming infertile	-	37(42.0%)	-
			Fear of interference with breastfeeding	-	34(38.6%)	-
			Family pressure	-	12(13.6%)	-
			No reason stated	-	5(5.8%)	-

Similarly, socioeconomic status also correlated with postpartum contraceptive acceptance (42.3% of contraception users belonged to lower socioeconomic status compared to 88.6% in the non-users group; p-value 0.003) (Table 1). Socio-economic status was classified according to the Modified Kuppuswamy Scale, which is generally used in this area of research. Among the contraception users, 70% had four or more antenatal visits, while only 7.9% of the non-users had antenatal visits in all trimesters during pregnancy (p-value 0.001). No difference was noted in the mode of delivery and antenatal complications suffered by women in both groups. Common antenatal complications included anemia, hypothyroidism, pregnancy-induced hypertension (PIH), gestational diabetes, and intrahepatic cholestasis of pregnancy (since this study is a subset of the previously published original study [1], so details of the antenatal complications are presented in a tabulated form in that study). Although the majority of the adolescent mothers had antenatal complications like anemia and PIH, there was no statistically significant difference between the contraceptive users and non-users. Reasons for non-acceptance of postpartum contraception were elicited in Group B; 42.0% had fear of becoming infertile, 38.6% feared that the use of contraceptives during the postpartum period would interfere with the quality of milk and breastfeeding, 13.6% had opposition from family members, and 5.8% did not mention any reason.

## Discussion

In developing countries, almost 21 million adolescent girls conceive every year, and almost 12 million deliver, and among that, 7.7 million are less than the age of 15 years [4]. Marriage during adolescent years exposes them to a high risk of teenage pregnancy and higher maternal mortality [5]. A lower-middle-income country like India contributes 20% of the global 1.2 billion adolescents, among which 1.5 million girls get married before the age of 18 years [6]. In our study, all 133 participants were married in their adolescence and their mean age at the time of marriage was 17.6±0.4 and 17.5±0.6 years in Group A and Group B, respectively. Teenage pregnancies are associated with feto-maternal complications, like preterm labour, low birth weight babies, and obstructed labour and an increased risk of maternal and neonatal mortality [7].

In our study, antenatal complications were noted in 86.7 % and 88.6 % of Group A and Group B, respectively. So it is crucial to insist and counsel regarding contraception in adolescent girls and it is of utmost need to insist on postpartum contraception in teenage primigravidae to prevent further pregnancies. Adolescents experience barriers to access to contraception in various ways like inconvenient medical consultation hours, financial reasons, lack of confidentiality, and lack of trained health professionals [8]. In our study, almost 77.2 % belonged to upper lower socioeconomic status in Group B. The Contraceptive CHOICE Project stated that the availability of free contraception services to adolescents can prevent teenage pregnancy, teenage delivery, and unsafe abortions [9]. Contraception services for adolescence are available free of cost in a few developing countries, but the awareness regarding this is poor. Poor educational status has been proposed to be a single factor resulting in poor knowledge and awareness regarding contraception [10].

Adolescents should be made aware of contraception usage in every possible situation. American College of Obstetricians & Gynecologists (ACOG) recommends that gynecologists should routinely address contraceptive needs and concerns irrespective of the patient’s age or previous sexual activity [11]. Adolescent reproductive and sexual health strategy was instituted to increase awareness and access to reproductive health services [12]. Besides poor awareness, there exist misconceptions about contraception resulting in under-usage of contraception [13].

In our study, almost 42 % of the non-users stated fear of becoming infertile as the reason for not using contraception which is a misconception. And in a few countries, particularly in lower-middle-income countries, there exists societal and familial pressure to conceive soon after marriage to prove fertility, thus hurdling contraceptive use [14]. In our study, almost 13.6 % of non-users had opposition from family members. The number of antenatal visits influences the use of postpartum contraception. Mothers who had four and more antenatal visits were three times more likely to follow postpartum contraception [15]. Among the contraception users, 70% had four or more than four antenatal visits, whereas only 7.9 % had four and more than four antenatal visits in non-users. Women who had caesarean delivery were 2.6 times more likely to accept and use postpartum contraception than those who had vaginal delivery [16]. This is contradictory to our study in which 60% of the contraception users had a vaginal delivery. This study underlines the importance of postpartum contraception in young mothers. It reiterates the role of education and socio-economic status in the acceptance of contraception. 

The study had a few limitations. It was a non-randomized single-center study. The limited sample size of the study was due to the lesser number of adolescent pregnancies in the modern scenario. Larger multicenter studies in varied ethnic populations would help to deduce better conclusions regarding contraceptive choices among teenage postpartum women.

## Conclusions

Teenage pregnancy is associated with increased feto-maternal complications. It also accounts for an increased incidence of unsafe abortions and maternal mortality. So it is crucial to make the adolescent group aware of contraception to prevent adolescent pregnancy. It is far more important to counsel regarding postpartum contraception to prevent further adolescent pregnancy. Larger multicentric studies with different ethnic groups may highlight the importance of interventions using educational and QI initiatives in improving the acceptance of contraception in such women. It is vital to create awareness through educational campaigns to increase the use of contraception and to decrease the incidence of teenage pregnancy.
